# Disrupted core-periphery structure of multimodal brain networks in Alzheimer’s disease

**DOI:** 10.1162/netn_a_00087

**Published:** 2019-05-01

**Authors:** Jeremy Guillon, Mario Chavez, Federico Battiston, Yohan Attal, Valentina La Corte, Michel Thiebaut de Schotten, Bruno Dubois, Denis Schwartz, Olivier Colliot, Fabrizio De Vico Fallani

**Affiliations:** Institut du Cerveau et de la Moelle Epiniere, ICM, Inserm, U 1127, CNRS, UMR 7225, Sorbonne Universite, Paris, France; Inria Paris, Aramis Project Team, Paris, France; CNRS, UMR 7225, Paris, France; Inria Paris, Aramis Project Team, Paris, France; CNRS, UMR 7225, Paris, France; Department of Network and Data Science, Central European University, Budapest, Hungary; MyBrain Technologies, Paris, France; Department of Neurology, Institute of Memory and Alzheimer’s Disease, Assistance Publique - Hopitaux de Paris, Pitié-Salpêtrière Hospital, Paris, France; Inserm, UMR 894, Center of Psychiatry and Neurosciences, Memory and Cognition Laboratory, Paris, France; Institute of Psychology, University Paris Descartes, Sorbonne Paris Cite, France; Institut du Cerveau et de la Moelle Epiniere, ICM, Inserm, U 1127, CNRS, UMR 7225, Sorbonne Universite, Paris, France; Institut de la Memoire et de la Maladie d’Alzheimer - IM2A, AP-HP, Sorbonne Universite, Paris, France; Institut du Cerveau et de la Moelle Epiniere, ICM, Inserm, U 1127, CNRS, UMR 7225, Sorbonne Universite, Ecole Normale Superieure, ENS, Centre MEG-EEG, Paris, France; Institut du Cerveau et de la Moelle Epiniere, ICM, Inserm, U 1127, CNRS, UMR 7225, Sorbonne Universite, Paris, France; Inria Paris, Aramis Project Team, Paris, France; Institut du Cerveau et de la Moelle Epiniere, ICM, Inserm, U 1127, CNRS, UMR 7225, Sorbonne Universite, Paris, France; Inria Paris, Aramis Project Team, Paris, France

**Keywords:** Neurodegenerative diseases, Brain connectivity, Multilayer network theory, MEG, DWI, fMRI

## Abstract

In Alzheimer’s disease (AD), the progressive atrophy leads to aberrant network reconfigurations both at structural and functional levels. In such network reorganization, the core and peripheral nodes appear to be crucial for the prediction of clinical outcome because of their ability to influence large-scale functional integration. However, the role of the different types of brain connectivity in such prediction still remains unclear. Using a multiplex network approach we integrated information from DWI, fMRI, and MEG brain connectivity to extract an enriched description of the core-periphery structure in a group of AD patients and age-matched controls. Globally, the regional coreness—that is, the probability of a region to be in the multiplex core—significantly decreased in AD patients as result of a random disconnection process initiated by the neurodegeneration. Locally, the most impacted areas were in the core of the network—including temporal, parietal, and occipital areas—while we reported compensatory increments for the peripheral regions in the sensorimotor system. Furthermore, these network changes significantly predicted the cognitive and memory impairment of patients. Taken together these results indicate that a more accurate description of neurodegenerative diseases can be obtained from the multimodal integration of neuroimaging-derived network data.

## Author Summary

Alzheimer’s disease includes a progressive destruction of axonal pathways leading to global network changes. While these changes affect both the anatomy and the function of the brain, a joint characterization of the impact on the nodes of the network is still lacking. By integrating information from multiple neuroimaging data, within a modern complex systems framework, we show that the nodes constituting the core of the brain network are the most impacted by the disconnection process. Furthermore, these network alterations significantly predict the cognitive and memory impairment of patients and represent potential biomarkers of disease progression. We posit that a more accurate description of neurodegenerative diseases can be obtained by analyzing and modeling brain networks derived from multimodal neuroimaging data.

## Introduction

The brain is a complex network where differently specialized areas are anatomically and functionally connected. Because of such interconnected structure, focal damages can affect the rest of the network through the interruption of communication pathways. Indeed, many neurological disorders affecting language, motor, and sensory abilities are often due to a disconnection syndrome caused by the anatomical connectivity breakdown between the relevant brain areas (Geschwind, 1965; Schmahmann & Pandya, 2008). In the case of neurodegenerative diseases, the disconnection hypothesis is supported by a progressive death of neurons and synapses that induce gross atrophy. Empirical evidence has shown that Alzheimer’s disease (AD) patients with severe motor and cognitive impairments exhibited anatomical disconnections among regions between cerebral hemispheres that resemble those observed in split-brain subjects (Delbeuck, Collette, & Van der Linden, 2007; Lakmache, Lassonde, Gauthier, Frigon, & Lepore, 1998). In Parkinson’s disease (PD) intrahemispheric dissociations between subcortical and cortical structures have been linked to disturbances in cognition, perception, emotion, and sleep (Cronin-Golomb, 2010). In addition, functional connectivity alterations within and between hemispheres have been reported in both AD (Adler, Brassen, & Jajcevic, 2003; Babiloni et al., 2009; Blinowska et al., 2016; Sankari, 2010) and PD (Dubbelink et al., 2013; Luo et al., 2015), suggesting their potential role in early diagnosis.

Altogether, these findings suggest that neurodegenerative diseases should be considered as a network problem. Recent approaches based on network theory have greatly advanced our understanding of the connection mechanisms characterizing brain diseases (Stam, 2014). Among others, decreased efficiency, modularity, and hub centrality have been largely reported in neurodegeneration and associated with the stage of disease. Increasing evidence suggests that the core-periphery structure of the human connectome—supporting global integration of information among distant areas—is highly affected by the AD process and that resulting changes might be effective predictors of cognitive declines. On one hand, brain areas forming the core of the network—that is, central and mutually connected nodes—have been reported to be preferentially attacked by AD (Yan et al., 2018). On the other hand, brain regions forming the periphery of the network—that is, nodes that are only weakly connected to the other units in the network—appear to be crucial for the degeneration (Daianu et al., 2015). While these results refer to structural brain connectivity, the relative contribution of functional brain connectivity into the network core-periphery changes remains poorly understood.

Based on the aforementioned empirical and theoretical grounds, we hypothesize that neurodegeneration would affect the core-periphery structure of the brain network at both anatomical and functional levels. More specifically, we expected that the extraction of the core-periphery organization by integrating information from multimodal brain networks would give more accurate predictors of AD and cognitive impairment. Finally, based on the evidence that hubs are the most attacked nodes in many neurological diseases and psychiatric disorders (van den Heuvel & Sporns, 2013), we hypothesize that the core brain regions would be mostly impacted by the AD atrophy process.

**Figure 1.**
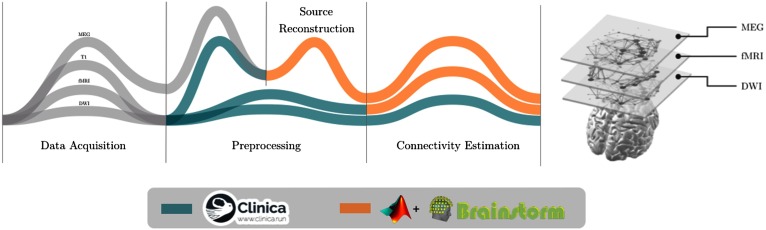
Multiplex brain network construction. Different neuroimaging data are collected and preprocessed separately. We used the Desikan cortical atlas parcellation (Desikan et al., 2006) to infer connectivity networks from DWI, fMRI, and MEG source-reconstructed data. The color of the line indicates the software that has been used in each step of the pipeline. We spatially aligned all the estimated brain networks to construct the multiplex brain network.

To test these predictions, we considered multiple brain networks derived from DWI, fMRI, and MEG data recorded in a group of AD patients and age-matched healthy controls (HC; Figure 1). Cognitive impairments in AD patients were described using multidomain behavioral measurements. We extracted the multimodal core-periphery structure of the brain networks through a multiplex network approach, where all the available information is kept at different connectivity layers. Multiplex network theory has been recently introduced to specifically model and analyze complex systems whose units can be linked through different types of connectivity (Battiston, Nicosia, & Latora, 2014; Boccaletti et al., 2014; De Domenico et al., 2013; Kivelä et al., 2014). Main applications in neuroscience have focused on the characterization of higher order network motifs (Battiston, Nicosia, Chavez, & Latora, 2017; Crofts, Forrester, & O’Dea, 2016; Pedersen, Zalesky, Omidvarnia, & Jackson, 2018) and node centrality (Bentley et al., 2016; De Domenico, Sasai, & Arenas, 2016; Guillon et al., 2017) in both healthy and diseased conditions (De Domenico et al., 2016; Guillon et al., 2017; Yu et al., 2017). Here, we evaluated how AD impacted the multiplex core-periphery organization (Battiston, Guillon, Chavez, Latora, & De Vico Fallani, 2018), and we tested the correlation of the regional coreness with the cognitive and memory impairment of patients. See the Material and Methods section for more details on the experimental design and methods of analysis.

## RESULTS

### Multimodal Core of Brain Networks

We integrated multimodal information by constructing nine-layer multiplex brain networks containing DWI, fMRI, and MEG connectivity between 68 cortical regions of interest (ROIs; Material and Methods). To estimate the likelihood of each ROI *i* to be in the multiplex core we computed its *coreness* 𝒞_*i*_ by counting how many times it was in the multiplex core across different density thresholds (Battiston et al., 2018). At each threshold, the multiplex core-periphery structure was obtained by linearly combining the node strength of all the layers through a vector parameter *c* (Material and Methods).

**Figure 2.**
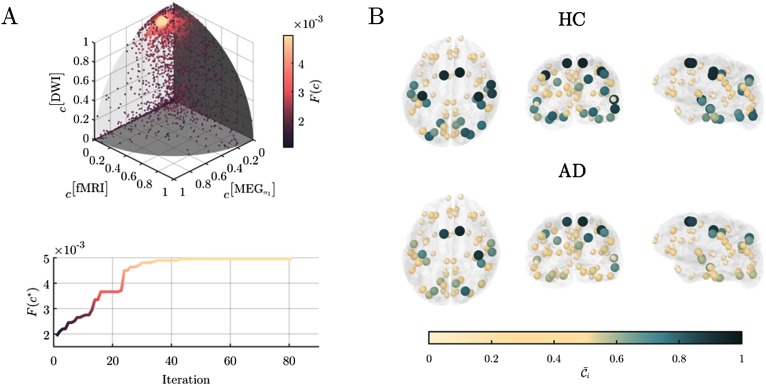
Regional coreness of the multiplex brain networks. Panel A), shows the results of the particle swarm optimization (PSO) used to find the best layer coefficients vector *c* that maximizes the Fisher score *F*(*c*) between AD and HC subjects. In the upper plot, each dot represents the position of a particle at a given iteration in the original 9-dimension coreness contribution coefficient vector space. The color of the dots code for the corresponding Fisher score. Results were projected over the three main network layers for the sake of illustration The other nonshown components were rapidly zeroing-out during the 81 iterations needed to converge to the optimum as shown in the bottom plot. Panel B) shows the****corresponding****average coreness for the healthy control (HC) population and for the Alzheimer’s disease (AD) group. The position of the nodes identifies the barycenter of each ROI in the cortical surface, here represented in transparent gray; the color of each node codes for the average coreness ${\bar{C}}_{i}$

Because we do not know a priori the best combination, we derived the optimal c^*^ by using a data-driven approach that efficiently explores the parameter space to maximize the difference between AD and HC regional coreness. Specifically, we used the particles swarm optimization algorithm (PSO) to maximize the Fisher’s criterion *F*(*c*) (Material and Methods). Results show that the optimal c^*^ components are found to be highly heterogenous and that the DWI layer, as well as MEG-alpha1 and fMRI layers, are the main contributors to separate the AD and HC group (Table 1, Figure 2A).

**Table 1.** Vector of the optimal layer weight for the coreness computation

Layer *m*	c^*[*m*]^
MEG_*δ*_	0.000
MEG_*θ*_	0.001
MEG_*α*_1__	0.258
MEG_*α*_2__	0.000
MEG_β_1__	0.000
MEG_*β*_2__	0.002
MEG_*γ*_	0.000
fMRI	0.104
DWI	0.961

In the HC group, the multiplex core tended to include large portions of temporal, superior parietal, and occipital cortices, and to a minor extent central and superior frontal regions (Figure 2B). On average AD patients exhibited a loss of coreness with respect to HC particularly in the temporal, superior parietal, and occipital cortices. These regions were already known to form the core of multiplex brain networks derived from DTI and fMRI data (Battiston et al., 2018).

### Reorganization of Core-Periphery Structure in AD

**Figure 3.**
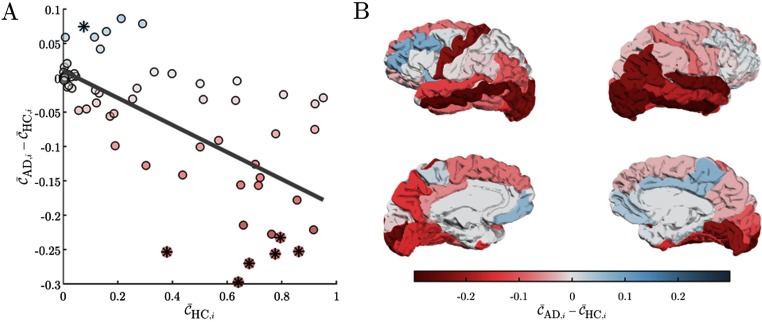
Differences in regional coreness between AD and HC subjects. Panel A) shows the between-group difference of coreness ${\bar{C}}_{\text{AD}},i$ − ${\bar{C}}_{\text{HC}},i$ as a function of the healthy population’s coreness ${\bar{C}}_{\text{HC}},i$; the slope of the regression line in gray measures the coreness disruption index *κ* = −0.20. The color of the circles code for the difference between average coreness in the AD and HC groups; stars point out the ROIs for which we reported a significant difference (*p* < 0.025, Supplementary Table S2; Guillon et al., 2019). Panel B) illustrates the values of the between-group coreness difference over the Desikan cortical atlas. Color code is the same as in panel A.

To quantify the observed network changes, we defined the coreness disruption index *κ* as the slope of the line obtained by regressing the difference between the average coreness (at each ROI and across subjects) of the two groups with the average coreness of the healthy one (Termenon, Achard, Jaillard, & Delon-Martin, 2016) (Material and Methods). We found a significant negative *κ* value, indicating that AD preferentially attack ROIs with a high coreness (*κ* = −0.20, *p* = 2.45e−10). This result was also consistent at the individual level when we extracted the coreness disruption index in each patient (Supplementary Table S1; Guillon et al., 2019). In particular, by statistically comparing the average coreness of the two groups, we reported a significant decrease of coreness in core regions, such as temporal, parietal, and occipital cortices as well as a significant increase of coreness in the right paracentral area that are instead more peripheral (*p* < 0.025, Figure 3A, B, Supplementary Table S2; Guillon et al., 2019).

**Figure 4.**
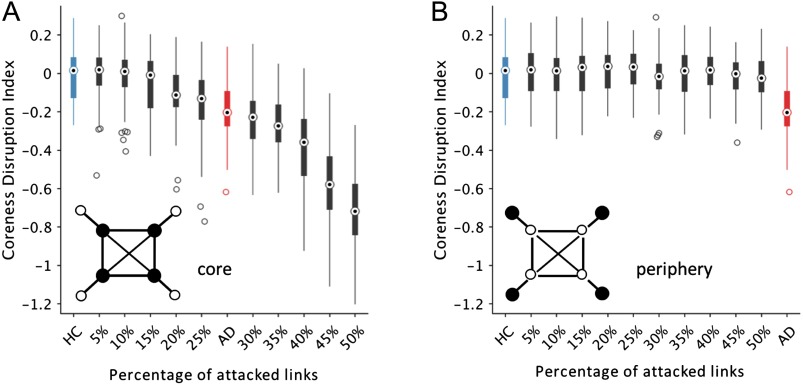
Coreness disruption index as a function of the network disconnection process. Boxplots show the values of coreness disruption index (*κ*) obtained by randomly attacking an increasing percentage of links in the multiplex brain networks of the HC group (Material and Methods). Panel A) shows the values obtained when the links connected to the multiplex core are preferentially attacked. Panel B) shows the values obtained when the links connected to the multiplex periphery are preferentially attacked. In both cases, the intensity of the selected links are decreased by 75%. The blue and red boxplots illustrate respectively the *κ* values for the HC and AD groups. The circles in the boxes show the median; the bottom and top edges of the boxes denote the 25th and 75th percentile, respectively. Whiskers connect the most extreme points not considered outliers, and outliers are plotted individually as circles.

Based on the hypothesis that AD is a disconnection syndrome (Delbeuck et al., 2007; Geschwind, 1965) leading to disorganized network configurations (Sanz-Arigita et al., 2010), we next generated a series of synthetic multiplex networks starting from the ones observed in the HC group and attacking an increasing amount of links in each layer. Specifically, we simulated the dysconnection process by decreasing the weights of the links that were preferentially connected to the core nodes (Materials and Methods). Results show that the coreness disruption index decreased with the number of links that were randomly attacked when the reduction was sufficiently strong, that is, 75%. In this situation, we could generate the same *κ* values observed in the multiplex brain networks of the AD group by attacking between 25% and 30% of the links (Figure 4A). Notably, this result could not be obtained when we attacked the links preferentially connected to peripheral nodes (Figure 4B). Altogether, these findings indicate that AD is associated with a pervasive random reconfiguration of brain connectivity that primarily affects the nodes of the multiplex core.

### Coreness Disruption Predicts Cognitive and Memory Deficits

**Figure 5.**
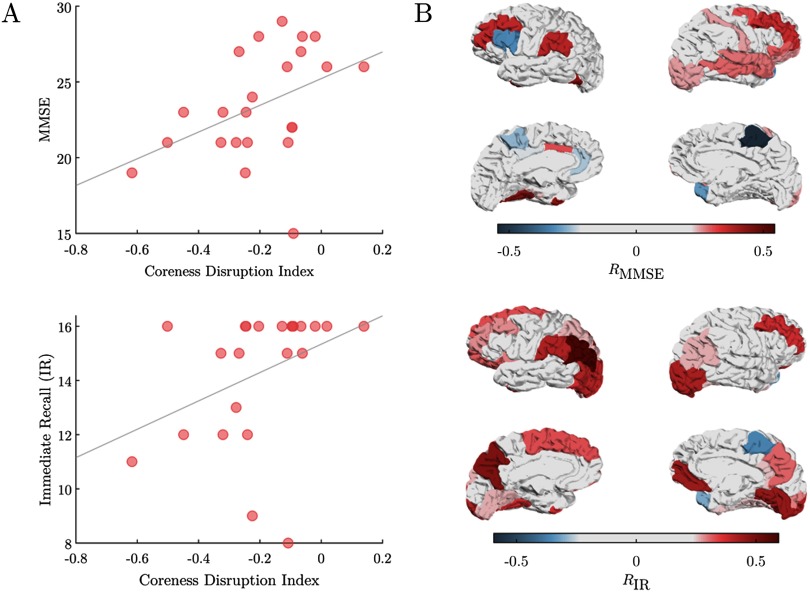
Correlation between coreness and cognitive/memory deficit. Panel A) shows****the values of the Mini–Mental State Examination (MMSE) and immediate recall (IR) as a function of the coreness disruption index *κ*. In panel B) the Spearman correlation values (R) between the regional coreness 𝒞_*i*_ and the MMSE and IR values are shown over the Desikan cortical atlas.

We finally conducted a correlation analysis to better understand how the observed multiplex brain network changes were associated with the behavioral performance of AD patients. Results show that both cognitive and memory deficits could be predicted by the individual loss of regional coreness. At the global scale, the coreness disruption index significantly correlated with the Mini–Mental State Examination (MMSE) (*R* = 0.46, *p* = 0.028) as well as with the immediate (*R* = 0.47, *p* = 0.024) and free recall (*R* = 0.59, *p* = 0.005) scores. The higher the *κ* values, the better was the performance of the patients (Figure 5A, Supplementary material; Guillon et al., 2019). At the local scale, temporal, parietal, and cortices were highly positively correlated with the behavior of patients. Notably, these ROIs overlapped with those exhibiting significant decreases of regional coreness with respect to healthy controls (Figure 3B). We found similar positive correlations for bilateral middle frontal ROIs (*R* = 0.36, *p* = 0.092 for left, *R* = 0.35, *p* = 0.100 for right), while areas in the motor system appeared not to be involved except for the paracentral lobule that tended to negatively correlate with the MMSE (*R* = −0.55, *p* = 0.007) and immediate recall scores (*R* = −0.36, *p* = 0.089; Figure 5B).

## DISCUSSION

### Multiplex Brain Networks

The increasing availability of multimodal neuroimaging data holds a great potential to enrich our knowledge about fundemental neural mechanisms and to improve the precision of predictive biomarkers of brain diseases (Calhoun & Sui, 2016). However, how to integrate information from different neuroimaging modalities is still an open issue. Existing approaches have mainly focused on merging information at the level of the native data structure (e.g., signal or images; Biessmann, Plis, Meinecke, Eichele, & Muller, 2011; Uludağ & Roebroeck, 2014). Only recently, investigators have started to propose fusion algorithms in an effort to infer brain connectivity (Ng, Varoquaux, Poline, & Thirion, 2012) or to detect mental states (Lei et al., 2011). Here, we adopted a complementary solution—based on the nascent field of multilayer networktheory—that preserves the original nature of the different connectivity types. Similar approaches have been already used in the case of temporal (Bassett et al., 2011; Pedersen et al., 2018), multifrequency (Brookes et al., 2016; De Domenico et al., 2016; Guillon et al., 2017; Tewarie et al., 2016; Yu et al., 2017), and DTI-fMRI brain networks (Battiston et al., 2017) in human but also in nonhuman species (Bentley et al., 2016; Crofts et al., 2016). This study considers for the first time brain networks obtained from three different neuroimaging modalities—DWI, fMRI, and MEG—to construct multiplex brain networks consisting of nine connectivity layers and to derive an augmented description of their core-periphery structure in healthy and Alzheimer’s diseased subjects. A crucial step in the characterization of multiplex networks is how to weight the contribution from different layers (Boccaletti et al., 2014; Kivelä et al., 2014), which typically contain connectivity measured in different units (e.g., number of fiber tracks and amount of signal correlation). While this is in general an arbitrary choice, here we established an objective way to associate a weight to each layer by maximizing the difference of regional coreness—that is, the likelihood of each region to be in the core—between the groups. Results showed that all three modalities are necessary to the group separation. In particular, for MEG only alpha1 was determinant while the other frequency layers had very low, or null, weights. This is in line with current evidence showing that the alpha1 frequency band contains the most discriminant power and connectivity changes in AD (Babiloni et al., 2004; Blinowska et al., 2016). Notably, DWI had a very high contribution coefficient as compared with the other layers. Core-periphery structure of diffusion-based networks is known to be very robust (Hagmann et al., 2008; van den Heuvel & Sporns, 2011) with respect to functional layers, and this might possibly depend on the heterogeneity of the weighted node degree distribution. More in general, further research is needed to derive criteria that objectively balance the layer contribution in the extraction of multiplex network properties.

### Network Reorganization in Alzheimer’s Disease

AD is associated with network changes affecting the structure and function of the brain at multiple spatial and temporal scales (Stam, 2014). It has been hypothesized that these network reconfigurations could result from dysconnection patterns initiated by the gross atrophy of the brain. While several studies have found significant changes in terms of network efficiency, modularity, and node centrality, the direction of these alterations—in terms of increments or decrements with respect to healthy controls—is often unclear and modality dependent (Tijms et al., 2013). Here, we focused on the core-periphery structure of the human brain, which has been shown to have a significant impact on cognition ensuring global integration across remote cortical areas (van den Heuvel & Sporns, 2011). Structural connectome studies have reported that AD patients, from the preclinical to dementia stages, have significant hub-concentrated lesion distributions (Brier et al., 2014; Buckner et al., 2009; Crossley et al., 2014; Dai et al., 2014; Shu, Wang, Bi, Zhao, & Han, 2018). However, recent evidence is suggesting that network disruption is prevalent in the peripheral network components in both AD (Daianu et al., 2015) and mild cognitive impairement (MCI) patients (Zhao et al., 2017). These inconsistent findings suggest that the network disruption mechanisms remain unclear. By integrating information from structural and functional brain networks, we aimed to overcome this controversy and provide a more comprehensive insight. Our multiplex network approach shows that core regions were globally affected in AD patients as compared with HC subjects and that this result could be modeled by a global random rewiring process. Specifically, we reported significant decrements of coreness in temporal and parietal cortices, which are heavily affected by atrophy processes and beta-amyloid deposition (Buckner, Andrews-Hanna, & Schacter, 2008). However, this change was paralleled by a significant increase of coreness in the paracentral lobules, which originally belonged to the multiplex periphery. Because regions of the sensorimotor system—such as paracentral lobule—are not directly affected by the atrophy process (Agosta et al., 2010), we speculate that possible compensatory mechanisms could have therefore taken place. In line with this hypothesis, recent findings suggest that more efficient motor commands in mild cognitive impaired patients could trigger the later functional decline (Kubicki, Fautrelle, Bourrelier, Rouaud, & Mourey, 2016). Longitudinal studies involving healthy subjects converting into AD will be fundamental to confirm or reject this prediction (Dubois et al., 2016).

### Connectivity-Based Biomarkers of Clinical Behavior

Brain wiring organization is critically associated with human cognition and behavior as well as with several neurological and psychiatric disorders (Stam, 2014). Network indices describing core-periphery and rich-club organization in structural brain networks have been shown to predict cognitive and motor deficits in multiple sclerosis (Stellmann et al., 2017), and Huntington disease (Harrington et al., 2015), as well as communication impairment in schizophrenia (van den Heuvel et al., 2013). More pertinent to this work, rich-club biomarkers extracted from DTI networks have been shown to correlate with cognitive and memory deficits in Alzheimer’s disease (Daianu et al., 2015; Stam et al., 2009; Tijms et al., 2013).

Here, we showed that the coreness disruption index—quantifying the global tendency to weaken core-periphery structure in multimodal brain networks—determined the cognitive and memory performance of our AD patients. Patients with a stronger core-periphery organization had higher MMSE and Free and Cued Selective Reminding Test (FCSRT) scores. At the local scale, temporal, parietal, as well as frontal areas tended to positively correlate with patients’ behavior. These association regions have been shown to be implicated in the prediction of AD cognitive performance (Khachiyants & Kim, 2012) and more in general in memory and language (Gordon, 1995; Pochon et al., 2002; Squire, 1992). We also found negative correlations with the paracentral lobule (especially right), a region that is typically involved in motor-related tasks but not in integrative functions.

From a network perspective, the coreness of regions that tended to be in the multiplex core—such as temporal, parietal, and occipital cortices—were positively correlated with patients’ performance, while among the peripheral areas the paracentral lobule was negatively correlated with the behavior. This means that in the presence of more severe cognitive and memory deficits, the relative decrease of connectivity in core regions tended to be replaced by periphery components of the brain system. This result would confirm the existence of an adjusting mechanism, where the sensorimotor system might be involved in the compensation of connectivity loss in systems that are directly impacted by amyloid-beta plaques and tau neurofibrillary tangles accumulation (Iaccarino et al., 2018).

### Methodological Considerations

The basic algorithm behind the detection of the core-periphery structure in multiplex networks is purely deterministic (Ma & Mondragón, 2015). This means that in principle we could not evaluate the statistical relevance of the identified structure. To overcome this limitation, we adopted a procedure that consisted in extracting the core-periphery structure from a series of multiplex networks obtained by filtering the actual brain multiplex network with increasing density thresholds (Battiston et al., 2018). This way we could derive a probabilistic measure of coreness by counting how many times each ROI was assigned to the core across all the possible thresholds. For the sake of simplicity we filtered each brain multiplex by retaining the strongest links so that the average node degree of each layer ranged from *k* = 1 to *k* = *N* − 1 (Material and Methods).

After filtering we did not binarize the surviving links so that we applied the core-periphery algorithm to sparse weighted multiplex networks. This approach allows us to exploit all the available information in the multiplex brain networks. At the same time, we remark that additional care is needed, as it introduces issues related to the different nature and distributions of the link weights (Buldú & Papo, 2018). Here, we mitigated this problem by using in the core-periphery algorithm the vector *c* of parameters that can weight the contribution of each layer (Material and Methods). Alternative solutions have been recently proposed taking into account the normalization of the weight across the layers by means of singular value decomposition (Mandke et al., 2018). Finally, because of the difficulty to measure connectivity between different neuroimaging modalities we did not consider interlayer links in our multiplex representation, as instead explored in more theoretical studies (Buldú & Porter, 2017; De Domenico et al., 2016). Despite the absence of interlinks, our multiplex network approach is able to extract higher order topological properties that cannot be obtained by other single-layer approaches. Notably, given the high level of overlap and correlations between the layers, our multiplex networks cannot be reduced to simple colored-edge graphs, where different colors are associated with different types of connectivity (Boccaletti et al., 2014).

We used an optimization algorithm—namely the particle swarm optimization—to find the best combination of *c* components that maximized the difference of the coreness between AD and HC subjects (Material and Methods). This method presents two limitations that are important to mention here. First, the time complexity increases exponentially with the number of layers *M* in order to find a stable solution. We verified that for *M* > 10 the research complexity becomes rapidly intractable because of the large space of parameter combination to explore. Second, the cost function optimized by the algorithm and used to evaluate how segregated the two groups are (i.e., two sets of coreness vectors) should be carefully chosen as its accuracy is highly impacted by the size of the feature space (here *N*, the number of ROIs) and the size of the samples (here the size of the cohorts). More advanced techniques taking into account the possible nonlinear and/or non-Euclidean nature of the feature space should be considered for very large networks (e.g., support vector machines, Riemannian geometry).

### Conclusion

Consistent with our hypothesis, we have shown that the AD atrophy process generates multimodal connectivity changes that can be quantified by a multilayer network approach. Specifically we have identified that both core and—to a minor extent—peripheral cortical areas are affected in AD, and that the direction of the effect was opposite. Decrease of coreness in temporal, parietal, and occipital areas—forming the rich core of the human brain—is paralleled by a possible compensatory increment in cortical regions that are in the sensorimotor system and that are more peripheral. These cortical network signatures varied over individuals and were significant predictors of cognitive and memory deficits. Furthermore, we reported a general framework for the statistical comparison of core-periphery organization in arbitrary multiplex networks. Taken together, our results offer new insights into the crucial role of core-periphery organization in neurodegenerative diseases.

## MATERIAL AND METHODS

### Cohort Inclusion

The study involved 23 Alzheimer’s diseased (AD) patients (13 women) and 26 healthy age-matched control (HC) subjects (19 women). All participants underwent the Mini–Mental State Examination (MMSE) for global cognition and the Free and Cued Selective Reminding Test (FCSRT) for verbal episodic memory. Inclusion criteria for all participants were (a) age between 50 and 90; (b) absence of general evolutive pathology; (c) no previous history of psychiatric diseases; (d) no contraindication to MRI examination; and (e) French as a mother tongue. Specific criteria for AD patients were (a) clinical diagnosis of Alzheimer’s disease; and (b) Mini–Mental State Examination (MMSE) score greater or equal to 18. All subjects gave written informed consent for participation in the study, which was approved by the local ethics committee of the Pitie-Salpetriere Hospital. All experiments were performed in accordance with relevant guidelines and regulation.

### Data Acquisition and Preprocessing

Magnetic resonance imaging (MRI) acquisitions were obtained using a 3T system (Siemens Trio, 32-channel system, with a 12-channel head coil). The MRI examination included (a) 3D T1-weighted volumetric magnetization-prepared rapid gradient echo (MPRAGE) sequence with the following parameters: thickness = 1 mm isotropic, repetition time (TR) = 2,300 ms, echo time (TE) = 4.18 ms, inversion time (TI) = 900 ms, acquisition matrix = 256 256; (b) echo planar imaging (EPI) sequence with the following parameters: one image with no diffusion sensitization (b0 image) and 50 diffusion-weighted images (DWI) at b = 1,500 s/mm^2^ , thickness = 2 mm isotropic, TR = 13,000 ms, TE = 92 ms, flip angle = 90, acquisition matrix = 128 116; (c) functional MRI (fMRI) resting-state sequence sensitive to blood oxygenation level-dependent (BOLD) contrast with the following parameters: 200 images, thickness = 3 mm isotropic, TR = 2,400 ms, TE = 30 ms, flip angle = 90, acquisition matrix = 64 64. All MR images were processed using the Clinica software (http://www.clinica.run). We first used the t1-freesurfer-cross-sectional pipeline to process T1-weighted images. This pipeline is a wrapper of different tools of the FreeSurfer software (http://surfer.nmr.mgh.harvard.edu/; Fischl, 2012). It includes segmentation of subcortical structures, extraction of cortical surfaces, cortical thickness estimation, spatial normalization onto the FreeSurfer surface template (FsAverage), and parcellation of cortical regions. Functional MRI images preprocessing has been conducted using the fmri-preprocessing pipeline. Slice timing correction, head motion correction, and unwarping have been applied using SPM12 tools (www.fil.ion.ucl.ac.uk/spm). Separately, the brain mask has been extracted from the T1 image of each subject using FreeSurfer. The resulting fMRI images have then been registered to the brain-masked T1 image of each subject using SPM’s registration tool. Finally, diffusion-weighted images have been processed using the dwi-preprocessing pipeline of Clinica. For each subject, all raw DWI volumes were rigidly registered (6 *df*) to the reference b0 image (DWI volume with no diffusion sensitization) to correct for head motion. The diffusion-weighting directions were appropriately updated (Leemans & Jones, 2009). An affine registration (12 *df*) was then performed between each DWI volume and the reference b0 to correct for eddy current distortions. These registrations were done using the FSL flirt tool (www.fmrib.ox.ac.uk/fsl). To correct for EPI-induced susceptibility artifacts, the field map image was used as proposed by Jezzard and Balaban (1995) with the FSL prelude/fugue tools. Finally, the DWI volumes were corrected for nonuniform intensity using the ANTs N4 bias correction algorithm (Tustison et al., 2010). A single multiplicative bias field from the reference b0 image was estimated, as suggested in Jeurissen, Tournier, Dhollander, Connelly, and Sijbers (2014).

The magnetoencephalography (MEG) experimental protocol consisted in a resting state with eyes closed (EC). Subjects were seated comfortably in a dimly lit electromagnetically and acoustically shielded room and were asked to relax. MEG signals were collected using a whole-head MEG system with 102 magnetometers and 204 planar gradiometers (Elekta Neuromag TRIUX MEG system) at a sampling rate of 1,000 Hz and online low-pass filtered at 330 Hz. The ground electrode was located on the right shoulder blade. An electrocardiogram (EKG, Ag/AgCl electrodes) was placed on the left abdomen for artifacts correction and a vertical electrooculogram (EOG) was simultaneously recorded. Four small coils were attached to the participant in order to monitor head position and to provide coregistration with the anatomical MRI. The physical landmarks (the nasion, the left and right preauricular points) were digitized using a Polhemus Fastrak digitizer (Polhemus, Colchester, VT). We extracted three consecutive clean epochs of approximately 2 min each.

Signal space separation was performed using MaxFilter to remove external noise. We used in-house software to remove cardiac and ocular blink artifacts from MEG signals by means of principal component analysis. We visually inspected the preprocessed MEG signals in order to remove epochs that still presented spurious contamination. At the end of the process, we obtained a coherent dataset consisting of three clean preprocessed epochs per subject. We reconstructed the MEG activity on the cortical surface by using a source imaging technique (Baillet et al., 2001; He, 1999): (a) We used the previously segmented T1-weighted images of each single subject (Fischl et al., 2002, 2004) to import cortical surfaces in the Brainstorm software (Tadel et al., 2011) where they were modeled with approximately 20,000 equivalent current dipoles (i.e., the vertices of the cortical meshes). (b) We applied the wMNE (weighted minimum norm estimate) algorithm with overlapping spheres (Lin et al., 2006) to solve the linear inverse problem. Both magnetometer and gradiometer, whose position has been registered on the T1 image using the digitized head points, were used to localize the activity over the cortical surface.

### Construction of Brain Networks

We built, for each modality, one or multiple brain connectivity networks whose nodes are regions of interest (ROIs) defined by the Desikan cortical atlas parcellation (Desikan et al., 2006; *N* = 68 regions); and links are weighted by a given connectivity measure estimated between each pair of nodes resulting in 68 × 68 fully symmetric adjacency matrices.

In the case of MEG, we used the spectral coherence as a connectivity estimator with the following parameters: window length = 2 s, window type = sliding Hanning, overlap = 25%, number of FFT points (NFFT) = 2,000 for a frequency resolution of 0.5 Hz between 2 Hz and 45 Hz included.

We then averaged the connectivity matrices within the following characteristic frequency bands (Babiloni et al., 2004; Stam et al., 2002): *delta* (2–4 Hz), *theta* (4.5–7.5 Hz), *alpha1* (8–10.5 Hz), *alpha2* (11–13 Hz), *beta1* (13.5–20 Hz), *beta2* (20.5–29.5 Hz), and *gamma* (30–45 Hz). We finally averaged the connectivity matrices across the three available epochs to obtain a robust estimate of the individual brain networks.

For fMRI data, we focused our analysis on the scale 2 wavelet correlation matrices that represented—with a TR = 2,400ms—the functional connectivity in the frequency interval 0.05–0.10Hz (Achard, 2006; Bassett & Bullmore, 2009; Biswal, Yetkin, Haughton, & Hyde, 1995; Cordes et al., 2001; De Vico Fallani, Richiardi, Chavez, & Achard, 2014). This choice was mainly motivated by the fact that the interpretation of different frequencies in fMRI is not clearly defined (Chen & Glover, 2015), whereas in E/MEG specific mental states can be more directly associated with distinct bands.

For DWI data, we used the Clinica software to estimate the fiber orientation distributions (FODs) using constrained spherical deconvolution (CSD) algorithm from MRtrix3 dwi2fod tool and tractography based on iFOD2 algorithm from MRtrix3 tckgen tool. The connectome is finally estimated by counting the number of tracts connecting each pair of nodes according to the given parcellation file using MRtrix3 tck2connectome tool.

### Network Methods and Models

We constructed multiplex brain networks in each subject by spatially aligning DWI, fMRI, and MEG source reconstructed connectivity networks. This led to the following multiplex network with *M* = 9 layers:

$$M=\left\{W^{\left[m\right]},\forall m\in \{{\text{MEG}}_{\delta },\ldots ,{\text{MEG}}_{\gamma },\text{fMRI},\text{DWI}\}\right\},$$ (1)

where *W*^[*m*]^ = {*w*_*ij*_^[*m*]^} is the connectivity matrix containing the weights of the connections between the ROIs *i* and *j* in the modality *m*. Because the weights in each layer can vary across different ranges, we applied the linear normalization $w_{ij}^{\left[m\right]}=\frac{w_{ij}^{\left[m\right]}\;-\;w_{min}^{\left[m\right]}}{w_{max}^{\left[m\right]}\;-\;w_{min}^{\left[m\right]}}$, where $w_{max}^{\left[m\right]}$ and $w_{min}^{\left[m\right]}$ are respectively the largest and smallest entries of *W*^[*m*]^. This way, all the links’ weights ranged between 0 and 1 and became comparable quantities across layers.

To extract the coreness of the nodes from the resulting multiplex networks, we followed the procedure described by Battiston et al. (2018). First, we filtered each layer by preserving the strongest weights for a broad range of increasing thresholds. Specifically, we considered density-based thresholds so that each layer had the same average node degree from *k* = 1 to *k* = *N* − 1. Then, for each threshold we computed the core-periphery of the filtered multiplex network by evaluating (a) the multiplex richness *μ*_*i*_ of node *i*, defined as follows:

$$\mu_{i}=\overset{M}{\underset{m=1}{\sum }}c^{\left[m\right]}s_{i}^{\left[m\right]},$$ (2)

with *s*_*i*_^[*m*]^ the strength of the node in the *m*-th layer, and *c*^[*m*]^ the components of the vector *c* that modulate the contribution of each modality-specific layer. And (b), similarly to the original paper, we decomposed the richness function into two components based on the links of node *i* that are going towards nodes with lower richness and those towards nodes with higher richness *s*^[*m*]^ = *s*^[*m*]−^ + *s*^[*m*] +^. Thus, the multiplex richness of a node towards richer nodes is defined as follows:

$$\mu_{i}^{+}=\overset{M}{\underset{m=1}{\sum }}c^{\left[m\right]}s_{i}^{[m]+}.$$ (3)

We finally counted the number of times that each node was in the core across all the explored thresholds, and we normalized by the maximum theoretical value. As a result, we obtained the coreness 𝒞_*i*_ that can be written as follows:

$$C_{i}=\frac{1}{N-1}\overset{N-1}{\underset{k=1}{\sum }}\delta_{i}^{\left[k\right]},$$ (4)

where

$$\delta_{i}^{\left[k\right]}=\left\{\begin{array}{cc} 1, & \text{if node}\;i\;\text{is in the core for the average node degree}\;k.\\ 0, & \text{otherwise}. \end{array}\right.$$

To simulate the disconnection process, we generated random multiplex networks by decreasing the intensity of the links in each layer starting from the actual multiplex brain network of the HC group. First, we fixed the percentage of links to be attacked and the percentage of weight reduction. Then, we randomly attacked the links by selecting those with higher probability to be connected to core or periphery nodes. For each HC individual, we generated *n*_rand_ new randomized multiplexes according to the following pseudocode:

▪ **Step 1**. Initialization
  (a)  Fix the number of links to randomly attack (*L*)
  (b)  Fix the percentage of weight reduction (*R*)
  (c)  Give a probability *p*(*i*) to each node proportional to its coreness
▪ **Step 2**. *Repeat until termination criteria are met* For *k* = 1, …, *L* do
  (a)  Pick a random node *i* with a probability *p*(*i*)
  (b)  Pick a random node *j* with a probability *p*(*j*)
  (c)  Decrease the weight of the link *w*_*ij*_ by *R*
  (d)  Exclude the link *i* − *j* in the next iterations
  (e)  *k* ← *k* + 1
▪ **Step 3**. Normalize and output connectivity matrix

We chose the minimum number of randomizations necessary to obtain a variance approximately equal to the one observed in the HC and AD groups. This number was *n*_rand_ = 3 and gave in total *N*_RA_ = *n*_rand_ × *N*_HC_ = 78 samples.

### Particles Swarm Optimization and Statistical Analysis

We used the PSO algorithm (Kennedy & Eberhart, 1995) under the MATLAB(R) software with the default parameters. The Fisher’s criterion *F*(*c*) was defined as follows:

$$F(c)=\frac{{\left({Ī}_{\text{AD}}(c)-{Ī}_{\text{HC}}(c)\right)}^{2}}{s_{\text{AD}}^{2}+s_{\text{HC}}^{2}},$$ (6)

with *Ī*_Pop_(*c*), the average local (i.e., node level) index, here the coreness 𝒞, over a population Pop, which in our case belongs to {AD, HC}, and,

$$s_{\text{Pop}}^{2}=\underset{s\in \text{Pop}}{\sum }{\left(I_{s}(c)-{Ī}_{\text{Pop}}(c)\right)}^{2},$$ (7)

with *s* a subject belonging to the population Pop.

Since, in our case, *F*(*c*) = *F*(*ac*),∀*a* ∈ℝ^ +^, and in order to save one dimension in the searching space, we expressed the coefficient *c* as a point on the positive section of the unitary hypersphere of dimension *M* = 9 such that

$$c=\left(\begin{array}{c} \sin\phi_{1}\ldots \text{sin}\phi_{8}\\ \sin\phi_{1}\ldots \text{sin}\phi_{7}\text{cos}\phi_{8}\\ \text{sin}\phi_{1}\ldots \text{sin}\phi_{6}\text{cos}\phi_{7}\\ \sin\phi_{1}\ldots \text{sin}\phi_{5}\text{cos}\phi_{6}\\ \text{sin}\phi_{1}\ldots \text{sin}\phi_{4}\text{cos}\phi_{5}\\ \text{sin}\phi_{1}\ldots \text{sin}\phi_{3}\text{cos}\phi_{4}\\ \text{sin}\phi_{1}\text{sin}\phi_{2}\text{cos}\phi_{3}\\ \text{sin}\phi_{1}\text{cos}\phi_{2}\\ \text{cos}\phi_{1} \end{array}\right),\phi_{k}\in [0,\frac{\pi }{2}],\forall k\in [1,M-1].$$

To consider the non-Gaussian nature of the data we considered nonparametric statistics when assessing differences between populations and prediction of behavioral scores. To these ends, we used respectively permutation *t* tests and Spearman correlation coefficients. The statistical threholds were set to *α* = 0.05, and we applied a rough false discovery rate (FDR) correction to account for the *N* = 68 post hoc tests at the level of brain regions (*α*_*FDR*_ = 0.025).

## AUTHOR CONTRIBUTIONS

Jeremy Guillon: Conceptualization; Data curation; Methodology; Software; Visualization; Writing - Original Draft; Writing - Review & Editing. Mario Chavez: Conceptualization; Methodology. Federico Battiston: Validation; Writing - Review & Editing. YohanAttal: Data curation. Valentina La Corte: Data curation. Michel Thiebaut de Schotten: Data curation; Writing - Review & Editing. Bruno Dubois: Funding acquisition; Project administration. Denis Schwartz: Data curation; Methodology; Resources; Validation; Writing - Review & Editing. Olivier Colliot: Funding acquisition; Project administration; Supervision. Fabrizio De Vico Fallani: Conceptualization; Methodology; Supervision; Validation; Visualization; Writing - Original Draft; Writing - Review & Editing.

## FUNDING INFORMATION

Fabrizio De Vico Fallani, Agence National de la Recherche, Award ID: ANR-15-NEUC-0006-02. Fabrizio De Vico Fallani, Agencenationale de la recherche, Award ID: ANR-10-IAIHU06. Bruno Dubois, Agencenationale de la recherche, Award ID: ANR-09-EMER-006. Olivier Colliot, Agencenationale de la recherche, Award ID: ANR-11-IDEX-004.

## TECHNICAL TERMS

Complex network: A collection of elements that interact dyadically in a nontrivial way.
Connectome: A network description of a neural system’s wiring; an exhaustive list of the physical connections (e.g., synapses, projections, fiber tracts) that link all neural elements (e.g., neurons, neuronal populations, macroscopic brain regions).
Multiplex network: A multilayer network where interlayer connections are allowed only between homologous nodes.
Multilayer network: A collection of elements that interact dyadically according to different types of connectivity. Each connectivity type is represented in a different layer, so that both intra- and interlayer connections are allowed.
Graph: A mathematical description of a network; elements and their interactions are represented as nodes/vertices and connections/edges, respectively.

## References

[bib1] Achard S. (2006). A resilient low-frequency, small-world human brain functional network with highly connected association cortical hubs. Journal of Neuroscience, 26(1), 63–72. 16399673 10.1523/JNEUROSCI.3874-05.2006 PMC6674299

[bib2] Adler G., Brassen S., & Jajcevic A. (2003). EEG coherence in Alzheimer’s dementia. Journal of Neural Transmission, 110(9), 1051–1058. 12928837 10.1007/s00702-003-0024-8

[bib3] Agosta F., Rocca M., Pagani E., Absinta M., Magnani G., Marcone A., … Filippi M. (2010). Sensorimotor network rewiring in mild cognitive impairment and Alzheimer’s disease. Human Brain Mapping, 31, 515–525. 19777557 10.1002/hbm.20883 PMC6871105

[bib4] Babiloni C., Binetti G., Cassetta E., Cerboneschi D., Dal Forno G., Del Percio C., … Rossini P. M. (2004). Mapping distributed sources of cortical rhythms in mild Alzheimer’s disease. A multicentric EEG study. NeuroImage, 22(1), 57–67. 15109997 10.1016/j.neuroimage.2003.09.028

[bib5] Babiloni C., Ferri R., Binetti G., Vecchio F., Frisoni G. B., Lanuzza B., … Rossini P. M. (2009). Directionality of EEG synchronization in Alzheimer’s disease subjects. Neurobiology of Aging, 30(1), 93–102. 17573161 10.1016/j.neurobiolaging.2007.05.007

[bib6] Baillet S., Riera J. J., Marin G., Mangin J. F., Aubert J., & Garnero L. (2001). Evaluation of inverse methods and head models for EEG source localization using a human skull phantom. Physics in Medicine and Biology, 46(1), 77–96. 11197680 10.1088/0031-9155/46/1/306

[bib7] Bassett D. S., & Bullmore E. T. (2009). Human brain networks in health and disease. Current Opinion in Neurology, 22(4), 340–347. 19494774 10.1097/WCO.0b013e32832d93dd PMC2902726

[bib8] Bassett D. S., Wymbs N. F., Porter M. A., Mucha P. J., Carlson J. M., & Grafton S. T. (2011). Dynamic reconfiguration of human brain networks during learning. PNAS, 108(18), 7641–7646. 21502525 10.1073/pnas.1018985108 PMC3088578

[bib9] Battiston F., Guillon J., Chavez M., Latora V., & De Vico Fallani F. (2018). Multiplex core–periphery organization of the human connectome. Journal of the Royal Society Interface, 15(146), 20180514 10.1098/rsif.2018.0514 PMC6170773 30209045

[bib10] Battiston F., Nicosia V., Chavez M., & Latora V. (2017). Multilayer motif analysis of brain networks. Chaos: An Interdisciplinary Journal of Nonlinear Science, 27(4), 047404 10.1063/1.4979282 28456158

[bib11] Battiston F., Nicosia V., & Latora V. (2014). Structural measures for multiplex networks. Physical Review E, 89(3), 032804 10.1103/PhysRevE.89.032804 24730896

[bib12] Bentley B., Branicky R., Barnes C. L., Chew Y. L., Yemini E., Bullmore E. T., … Schafer W. R. (2016). The multilayer connectome of Caenorhabditis elegans. PLoS Computational Biology, 12(12), e1005283 27984591 10.1371/journal.pcbi.1005283 PMC5215746

[bib13] Biessmann F., Plis S., Meinecke F. C., Eichele T., & Muller K.-R. (2011). Analysis of multimodal neuroimaging data. IEEE Reviews in Biomedical Engineering, 4, 26–58. 22273790 10.1109/RBME.2011.2170675

[bib14] Biswal B., Yetkin F. Z., Haughton V. M., & Hyde J. S. (1995). Functional connectivity in the motor cortex of resting human brain using echo-planar MRI. Magnetic Resonance in Medicine, 34(4), 537–541. 8524021 10.1002/mrm.1910340409

[bib15] Blinowska K. J., Rakowski F., Kaminski M., De Vico Fallani F., Del Percio C., Lizio R., & Babiloni C. (2016). Functional and effective brain connectivity for discrimination between Alzheimer’s patients and healthy individuals: A study on resting state EEG rhythms. Clinical Neurophysiology. 10.1016/j.clinph.2016.10.002 27836429

[bib16] Boccaletti S., Bianconi G., Criado R., del Genio C. I., Gómez-Gardeñes J., Romance M., … Zanin M. (2014). The structure and dynamics of multilayer networks. Physics Reports, 544(1), 1–122. 10.1016/j.physrep.2014.07.001 PMC7332224 32834429

[bib17] Brier M. R., Thomas J. B., Fagan A. M., Hassenstab J., Holtzman D. M., Benzinger T. L., … Ances B. M. (2014). Functional connectivity and graph theory in preclinical Alzheimer’s disease. Neurobiology of Aging, 35(4), 757–768. 24216223 10.1016/j.neurobiolaging.2013.10.081 PMC3880636

[bib18] Brookes M. J., Tewarie P. K., Hunt B. A. E., Robson S. E., Gascoyne L. E., Liddle E. B., … Morris P. G. (2016). A multi-layer network approach to MEG connectivity analysis. NeuroImage, 132, 425–438. 26908313 10.1016/j.neuroimage.2016.02.045 PMC4862958

[bib19] Buckner R. L., Andrews-Hanna J. R., & Schacter D. L. (2008). The brain’s default network. Annals of the New York Academy of Sciences, 1124(1), 1–38. 18400922 10.1196/annals.1440.011

[bib20] Buckner R. L., Sepulcre J., Talukdar T., Krienen F. M., Liu H., Hedden T., … Johnson K. A. (2009). Cortical hubs revealed by intrinsic functional connectivity: Mapping assessment of stability, and relation to Alzheimer’s disease. Journal of Neuroscience, 29(6), 1860–1873. 19211893 10.1523/JNEUROSCI.5062-08.2009 PMC2750039

[bib21] Buldú J. M., & Papo D. (2018). Can multilayer brain networks be a real step forward? Physics of Life Reviews, 24, 153–155 . 29290618 10.1016/j.plrev.2017.12.007

[bib22] Buldú J. M., & Porter M. A. (2017). Frequency-based brain networks: From a multiplex framework to a full multilayer description. Network Neuroscience, 2(4), 418–441. 10.1162/netn_a_00033 PMC6147638 30294706

[bib23] Calhoun V. D., & Sui J. (2016). Multimodal fusion of brain imaging data: A key to finding the missing link(s) in complex mental illness. Biological Psychiatry: Cognitive Neuroscience and Neuroimaging, 1(3), 230–244. 27347565 10.1016/j.bpsc.2015.12.005 PMC4917230

[bib24] Chen J. E., & Glover G. H. (2015). BOLD fractional contribution to resting-state functional connectivity above 0.1 Hz. NeuroImage, 107, 207–218. 25497686 10.1016/j.neuroimage.2014.12.012 PMC4318656

[bib25] Cordes D., Haughton V., Arfanakis K., Carew J., Turski P., Moritz C., … Meyerand M. (2001). Frequencies contributing to functional connectivity in the cerebral cortex in resting-state data. AJNR American Journal of Neuroradiology, 22, 1326–1333. 11498421 PMC7975218

[bib26] Crofts J. J., Forrester M., & O’Dea R. D. (2016). Structure-function clustering in multiplex brain networks. EPL, 116(1), 18003 10.1038/s41598-022-19994-9 PMC9537289 36202837

[bib27] Cronin-Golomb A. (2010). Parkinson’s disease as a disconnection syndrome. Neuropsychology Review, 20(2), 191–208. 20383586 10.1007/s11065-010-9128-8 PMC2882524

[bib28] Crossley N. A., Mechelli A., Scott J., Carletti F., Fox P. T., McGuire P., & Bullmore E. T. (2014). The hubs of the human connectome are generally implicated in the anatomy of brain disorders. Brain, 137(8), 2382–2395. 25057133 10.1093/brain/awu132 PMC4107735

[bib29] Dai Z., Yan C., Li K., Wang Z., Wang J., Cao M., … He Y. (2014). Identifying and mapping connectivity patterns of brain network hubs in Alzheimer’s disease. Cerebral Cortex, 25(10), 3723–3742. 25331602 10.1093/cercor/bhu246

[bib30] Daianu M., Jahanshad N., Nir T. M., Jack C. R., Weiner M. W., Bernstein M. A., & Thompson P. M. (2015). Rich club analysis in the Alzheimer’s disease connectome reveals a relatively undisturbed structural core network. Human Brain Mapping, 36(8), 3087–3103. 26037224 10.1002/hbm.22830 PMC4504816

[bib31] De Domenico M., Sasai S., & Arenas A. (2016). Mapping multiplex hubs in human functional brain networks. Frontiers in Neuroscience, 10 10.3389/fnins.2016.00326 PMC4945645 27471443

[bib32] De Domenico M., Solé-Ribalta A., Cozzo E., Kivelä M., Moreno Y., Porter M. A., … Arenas A. (2013). Mathematical formulation of multilayer networks. Physical Review X, 3(4), 041022

[bib33] Delbeuck X., Collette F., & Van der Linden M. (2007). Is Alzheimer’s disease a disconnection syndrome? Neuropsychologia, 45(14), 3315–3323. 17765932 10.1016/j.neuropsychologia.2007.05.001

[bib34] Desikan R. S., Ségonne F., Fischl B., Quinn B. T., Dickerson B. C., Blacker D., … Killiany R. J. (2006). An automated labeling system for subdividing the human cerebral cortex on MRI scans into gyral based regions of interest. NeuroImage, 31(3), 968–980. 16530430 10.1016/j.neuroimage.2006.01.021

[bib35] De Vico Fallani F., Richiardi J., Chavez M., & Achard S. (2014). Graph analysis of functional brain networks: Practical issues in translational neuroscience. Philosophical Transactions of the Royal Society of London B, 369(1653), 20130521 10.1098/rstb.2013.0521 PMC4150298 25180301

[bib36] Dubbelink K. T. E. O., Hillebrand A., Stoffers D., Deijen J. B., Twisk J. W. R., Stam C. J., & Berendse H. W. (2013). Disrupted brain network topology in Parkinson’s disease: A longitudinal magnetoencephalography study. Brain, 137(1), 197–207. 24271324 10.1093/brain/awt316

[bib37] Dubois B., Hampel H., Bakardjian H., Nyasse F., Lista S., Lamari F., … Habert M.-O. (2016). INSIGHT-AD study: A monocentric cohort for the study of the preclinical stage of Alzheimer’s disease. Alzheimer’s & Dementia, 12(7), P335

[bib38] Fischl B. (2012). FreeSurfer. NeuroImage, 62(2), 774–781. 22248573 10.1016/j.neuroimage.2012.01.021 PMC3685476

[bib39] Fischl B., Salat D. H., Busa E., Albert M., Dieterich M., Haselgrove C., … Dale A. M. (2002). Whole brain segmentation: Automated labeling of neuroanatomical structures in the human brain. Neuron, 33(3), 341–355. 11832223 10.1016/s0896-6273(02)00569-x

[bib40] Fischl B., Salat D. H., van der Kouwe A. J. W., Makris N., Ségonne F., Quinn B. T., & Dale A. M. (2004). Sequence-independent segmentation of magnetic resonance images. NeuroImage, 23(Suppl. 1), S69–S84. 15501102 10.1016/j.neuroimage.2004.07.016

[bib41] Geschwind N. (1965). Disconnexion syndromes in animals and man. Neuropsychology Review, 20(2), 128–157. 10.1007/s11065-010-9131-0 20540177

[bib42] Gordon B. (1995). Memory amnesia, and the hippocampal system. Electroencephalography and Clinical Neurophysiology, 95(6), 479

[bib43] Guillon J., Attal Y., Colliot O., Corte V. L., Dubois B., Schwartz D., … De Vico Fallani F. (2017). Loss of brain inter-frequency hubs in Alzheimer’s disease. Scientific Reports, 7(1), 10879 28883408 10.1038/s41598-017-07846-w PMC5589939

[bib44] Guillon J., Chavez M., Battiston F., Attal Y., La Corte V., Thiebaut de Schotten M., … De VicoFallani F. (2019). Supporting information for “Disrupted core-periphery structure of multimodal brain networks in Alzheimers disease.” Network Neuroscience, 3(2), 635–652. 10.1162/netn_a_00087 PMC6542619 31157313

[bib45] Hagmann P., Cammoun L., Gigandet X., Meuli R., Honey C. J., Wedeen V. J., & Sporns O. (2008). Mapping the structural core of human cerebral cortex. PLoS Biology, 6(7), e159 18597554 10.1371/journal.pbio.0060159 PMC2443193

[bib46] Harrington D. L., Rubinov M., Durgerian S., Mourany L., Reece C., Koenig K., … Rao S. M. (2015). Network topology and functional connectivity disturbances precede the onset of Huntington’s disease. Brain, 138(8), 2332–2346. 26059655 10.1093/brain/awv145 PMC5022662

[bib47] He B. (1999). Brain electric source imaging: Scalp Laplacian mapping and cortical imaging. Critical Reviews in Biomedical Engineering, 27(3–5), 149–188. 10864279

[bib48] Iaccarino L., Tammewar G., Ayakta N., Baker S., Bejanin A., Boxer A., … Rabinovici G. (2018). Local and distant relationships between amyloid, tau and neurodegeneration in Alzheimer’s disease. NeuroImage: Clinical, 17, 452–464. 29159058 10.1016/j.nicl.2017.09.016 PMC5684433

[bib49] Jeurissen B., Tournier J.-D., Dhollander T., Connelly A., & Sijbers J. (2014). Multi-tissue constrained spherical deconvolution for improved analysis of multi-shell diffusion MRI data. NeuroImage, 103, 411–426. 25109526 10.1016/j.neuroimage.2014.07.061

[bib50] Jezzard P., & Balaban R. S. (1995). Correction for geometric distortion in echo planar images from B0 field variations. Magnetic Resonance in Medicine, 34(1), 65–73. 7674900 10.1002/mrm.1910340111

[bib51] Kennedy J., & Eberhart R. (1995). Particle swarm optimization. In Proceedings of ICNN’95 - International Conference on Neural Networks. IEEE

[bib52] Khachiyants N., & Kim K. Y. (2012). Mini-mental status exam mapping to the corresponding brain areas in dementia. Applied Technologies and Innovations, 7(2), 55–58.

[bib53] Kivelä M., Arenas A., Barthelemy M., Gleeson J. P., Moreno Y., & Porter M. A. (2014). Multilayer networks. Journal of Complex Networks, 2(3), 203–271.

[bib54] Kubicki A., Fautrelle L., Bourrelier J., Rouaud O., & Mourey F. (2016). The early indicators of functional decrease in mild cognitive impairment. Frontiers in Aging Neuroscience, 8 10.3389/fnagi.2016.00193 PMC4981593 27570509

[bib55] Lakmache Y., Lassonde M., Gauthier S., Frigon J.-Y., & Lepore F. (1998). Interhemispheric disconnection syndrome in Alzheimer’s disease. Proceedings of the National Academy of Sciences, 95(15), 9042–9046. 10.1073/pnas.95.15.9042 PMC21199 9671801

[bib56] Leemans A., & Jones D. K. (2009). The B-matrix must be rotated when correcting for subject motion in DTI data. Magnetic Resonance in Medicine, 61(6), 1336–1349. 19319973 10.1002/mrm.21890

[bib57] Lei X., Ostwald D., Hu J., Qiu C., Porcaro C., Bagshaw A. P., & Yao D. (2011). Multimodal functional network connectivity: An EEG-fMRI fusion in network space. PLoS ONE, 6(9), e24642 21961040 10.1371/journal.pone.0024642 PMC3178514

[bib58] Lin F.-H., Witzel T., Ahlfors S. P., Stufflebeam S. M., Belliveau J. W., & Hämäläinen M. S. (2006). Assessing and improving the spatial accuracy in MEG source localization by depth-weighted minimum-norm estimates. NeuroImage, 31(1), 160–171. 16520063 10.1016/j.neuroimage.2005.11.054

[bib59] Luo C., Guo X., Song W., Zhao B., Cao B., Yang J., … Shang H.-F. (2015). Decreased resting-state interhemispheric functional connectivity in Parkinson’s disease. BioMed Research International, 2015, 1–8. 10.1155/2015/692684 PMC4477209 26180807

[bib60] Ma A., & Mondragón R. J. (2015). Rich-cores in networks. PLoS ONE, 10(3), e0119678 25799585 10.1371/journal.pone.0119678 PMC4370710

[bib61] Mandke K., Meier J., Brookes M. J., O’Dea R. D., Mieghem P. V., Stam C. J., … Tewarie P. (2018). Comparing multilayer brain networks between groups: Introducing graph metrics and recommendations. NeuroImage, 166, 371–384. 29138088 10.1016/j.neuroimage.2017.11.016

[bib62] Ng B., Varoquaux G., Poline J.-B., & Thirion B. (2012). A novel sparse graphical approach for multimodal brain connectivity inference. In Medical Image Computing and Computer-Assisted Intervention – MICCAI 2012 (707–714). Springer Berlin Heidelberg 10.1007/978-3-642-33415-3_87 23285614

[bib63] Pedersen M., Zalesky A., Omidvarnia A., & Jackson G. D. (2018). Multilayer network switching rate predicts brain performance. Proceedings of the National Academy of Sciences, 115(52), 13376–13381. 10.1073/pnas.1814785115 PMC6310789 30545918

[bib64] Pochon J. B., Levy R., Fossati P., Lehericy S., Poline J. B., Pillon B., … Dubois B. (2002). The neural system that bridges reward and cognition in humans: An fMRI study. Proceedings of the National Academy of Sciences, 99(8), 5669–5674. 10.1073/pnas.082111099 PMC122829 11960021

[bib65] Sankari Z. T. (2010). Local and distal coherence as a measure of cortical connectivity in Alzheimer’s disease. Alzheimer’s & Dementia, 6(4), S373

[bib66] Sanz-Arigita E. J., Schoonheim M. M., Damoiseaux J. S., Rombouts S. A. R. B., Maris E., Barkhof F., … Stam C. J. (2010). Loss of “small-world” networks in Alzheimer’s disease: Graph analysis of fMRI resting-state functional connectivity. PloS ONE, 5(11), e13788 21072180 10.1371/journal.pone.0013788 PMC2967467

[bib67] Schmahmann J., & Pandya D. (2008). Disconnection syndromes of basal ganglia thalamus, and cerebrocerebellar systems. Cortex, 44(8), 1037–1066. 18614161 10.1016/j.cortex.2008.04.004 PMC3738020

[bib68] Shu N., Wang X., Bi Q., Zhao T., & Han Y. (2018). Disrupted topologic efficiency of white matter structural connectome in individuals with subjective cognitive decline. Radiology, 286(1), 229–238. 28799862 10.1148/radiol.2017162696

[bib69] Squire L. R. (1992). Memory and the hippocampus: A synthesis from findings with rats, monkeys, and humans: Correction. Psychological Review, 99(3), 582 10.1037/0033-295x.99.2.195 1594723

[bib70] Stam C. J. (2014). Modern network science of neurological disorders. Nature Reviews Neuroscience, 15(10), 683–695. 25186238 10.1038/nrn3801

[bib71] Stam C. J., de Haan W., Daffertshofer A., Jones B. F., Manshanden I., van Cappellen van Walsum A. M., … Scheltens P. (2009). Graph theoretical analysis of magnetoencephalographic functional connectivity in Alzheimer’s disease. Brain, 132(1), 213–224. 18952674 10.1093/brain/awn262

[bib72] Stam C. J., van Cappellen van Walsum A. M., Pijnenburg Y. A. L., Berendse H. W., de Munck J. C., Scheltens P., & van Dijk B. W.(2002). Generalized synchronization of MEG recordings in Alzheimer’s disease: Evidence for involvement of the gamma band. Journal of Clinical Neurophysiology, 19(6), 562–574. 12488788 10.1097/00004691-200212000-00010

[bib73] Stellmann J.-P., Hodecker S., Cheng B., Wanke N., Young K. L., Hilgetag C., … Siemonsen S. (2017). Reduced rich-club connectivity is related to disability in primary progressive MS. Neurology - Neuroimmunology Neuroinflammation, 4(5), e375 10.1212/NXI.0000000000000375 PMC5532749 28804744

[bib74] Tadel F., Baillet S., Mosher J. C., Pantazis D., Leahy R. M., Tadel F., … Leahy R. M. (2011). Brainstorm: A user-friendly application for MEG/EEG analysis. Computational Intelligence and Neuroscience, 2011, e879716 10.1155/2011/879716 PMC3090754 21584256

[bib75] Termenon M., Achard S., Jaillard A., & Delon-Martin C. (2016). The “hub disruption index,” a reliable index sensitive to the brain networks reorganization. A study of the contralesional hemisphere in stroke. Frontiers in Computational Neuroscience, 10 10.3389/fncom.2016.00084 PMC4987351 27582702

[bib76] Tewarie P., Hillebrand A., van Dijk B. W., Stam C. J., O’Neill G. C., Van Mieghem P., … Brooks M. J. (2016). Integrating cross-frequency and within band functional networks in resting-state MEG: A multi-layer network approach. NeuroImage, 142, 324–336. 27498371 10.1016/j.neuroimage.2016.07.057

[bib77] Tijms B. M., Wink A. M., de Haan W., van der Flier W. M., Stam C. J., Scheltens P., & Barkhof F. (2013). Alzheimer’s disease: Connecting findings from graph theoretical studies of brain networks. Neurobiology of Aging, 34(8), 2023–2036. 23541878 10.1016/j.neurobiolaging.2013.02.020

[bib78] Tustison N. J., Avants B. B., Cook P. A., Zheng Y., Egan A., Yushkevich P. A., & Gee J. C. (2010). N4ITK: Improved N3 bias correction. IEEE Transactions on Medical Imaging, 29(6), 1310–1320. 20378467 10.1109/TMI.2010.2046908 PMC3071855

[bib79] Uludağ K., & Roebroeck A. (2014). General overview on the merits of multimodal neuroimaging data fusion. NeuroImage, 102, 3–10. 24845622 10.1016/j.neuroimage.2014.05.018

[bib80] van den Heuvel M. P., & Sporns O. (2011). Rich-club organization of the human connectome. Journal of Neuroscience, 31(44), 15775–15786. 22049421 10.1523/JNEUROSCI.3539-11.2011 PMC6623027

[bib81] van den Heuvel M. P., & Sporns O. (2013). Network hubs in the human brain. Trends in Cognitive Sciences, 17(12), 683–696. 24231140 10.1016/j.tics.2013.09.012

[bib82] van den Heuvel M. P., Sporns O., Collin G., Scheewe T., Mandl R. C. W., Cahn W., … Kahn R. S. (2013). Abnormal rich club organization and functional brain dynamics in schizophrenia. JAMA Psychiatry, 70(8), 783 23739835 10.1001/jamapsychiatry.2013.1328

[bib83] Yan T., Wang W., Yang L., Chen K., Chen R., & Han Y. (2018). Rich club disturbances of the human connectome from subjective cognitive decline to Alzheimer’s disease. Theranostics, 8(12), 3237–3255. 29930726 10.7150/thno.23772 PMC6010989

[bib84] Yu M., Engels M. M. A., Hillebrand A., van Straaten E. C. W., Gouw A. A., … Stam C. J. (2017). Selective impairment of hippocampus and posterior hub areas in Alzheimer’s disease: An MEG-based multiplex network study. Brain, 140(5), 1466–1485. 28334883 10.1093/brain/awx050

[bib85] Zhao T., Sheng C., Bi Q., Niu W., Shu N., & Han Y. (2017). Age-related differences in the topological efficiency of the brain structural connectome in amnestic mild cognitive impairment. Neurobiology of Aging, 59, 144–155. 28882420 10.1016/j.neurobiolaging.2017.08.005

