# Supplementary material for: Disrupted core-periphery structure of multimodal brain networks in Alzheimer’s disease
Source: Netw Neurosci. 2019 May 1;3(2):635–52. doi: 10.1162/netn_a_00087 (PMC6542619; doi:10.1162/netn_a_00087)
Supplement: Supplementary file 1 [file netn-03-635-s001.pdf]

Guillon, J., Chavez, M., Battiston, F., Attal, Y., La Corte, V., Thiebaut de Schotten, M., Dubois, B., Schwartz, D., Colliot, O., & De Vico Fallani, F. (2019). Supporting information for "Disrupted coreperiphery structure of multimodal brain networks 2 in Alzheimer's Disease." *Network Neuroscience*, *3*(2), 635-652. https://doi.org/10.1162/netn_a_00087

Disrupted core-periphery structure of multimodal brain networks in Alzheimer's Disease (Supplementary Material)

Jeremy Guillon[1,2], Mario Chavez[3], Federico Battiston[10,3,2], Yohan Attal[4], Valentina La Corte[5,6,7], Michel Thiebaut de Schotten[1], Bruno Dubois[8], Denis Schwartz[9], Olivier Colliot[2,1], and Fabrizio De Vico Fallani[2,1,*]

[1]Institut du Cerveau et de la Moelle Epiniere, ICM, Inserm, U 1127, CNRS, UMR 7225, Sorbonne Universite, F-75013, Paris, France

[2]Inria Paris, Aramis project-team, F-75013, Paris, France

[3]CNRS, UMR 7225, F-75013, Paris, France

[4]MyBrain Technologies, Paris, France

[5]Department of Neurology, Institut de la Memoire et de la Maladie d'Alzheimer - IM2A, Paris, France

[6]INSERM UMR 894, Center of Psychiatry and Neurosciences, Memory and Cognition Laboratory, Paris, France

[7]Institute of Psychology, University Paris Descartes, Sorbonne Paris Cite, France

[8]Institut de la Mémoire et de la Maladie d'Alzheimer - IM2A, AP-HP, Sorbonne Université, Paris, France

[9]Institut du Cerveau et de la Moelle Epiniere, ICM, Inserm U 1127, CNRS UMR 7225, Sorbonne Universite, Ecole Normale Superieure, ENS, Centre MEG-EEG, F-75013, Paris, France

[10]Department of Network and Data Science, Central European University, Budapest 1051, Hungary

[*]Corresponding author

December 7, 2018

Supplementary material

Supplementary figure

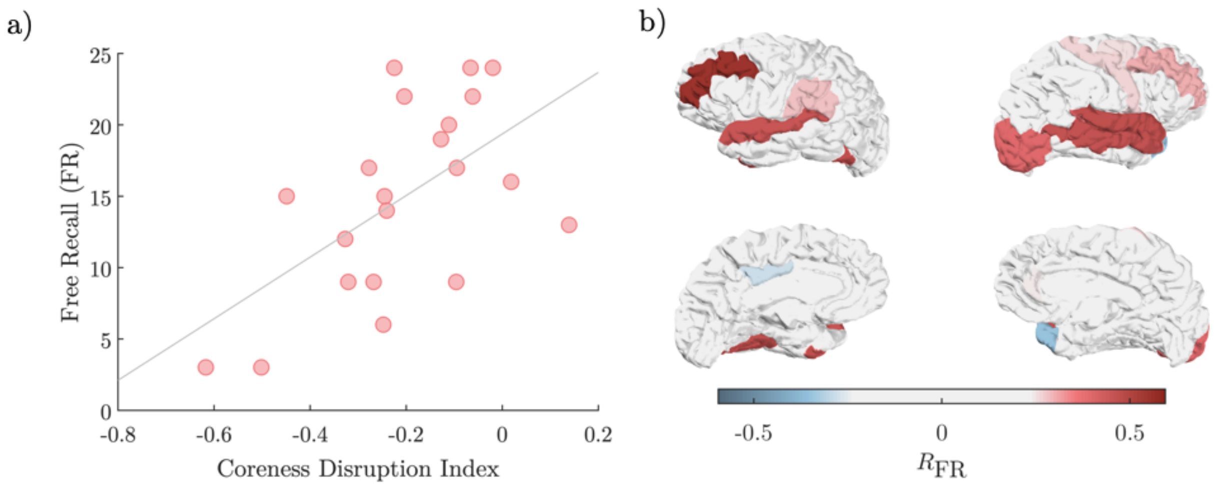

Figure S1: **Correlation between corenes and cognitive/memory deficit.** Panel **a)** shows the values of the free recall (FR) as a function of the coreness diruption index κ . In panel **b)** the Spearman correlation values (R_{FR}) between the regional coreness $\mathcal{C}_i$ and the FR values are shown over the Desikan cortical atlas.

Supplementary tables

Table S1: ADs population coreness disruption indices κ and the associated R^2 of the linear regression.

Subject ID	κ	R^2
1	-0,095	0,018
2	-0,111	0,033
3	-0,096	0,019
4	0,139	0,040
5	-0,502	0,412
6	0,018	0,001
7	-0,327	0,218
8	-0,617	0,267
9	-0,241	0,106
10	-0,062	0,015
11	-0,204	0,099
12	-0,245	0,085
13	-0,225	0,056
14	-0,020	0,001
15	-0,128	0,019
16	-0,248	0,141
17	-0,268	0,106
18	-0,278	0,081
19	-0,449	0,387
20	-0,091	0,011
21	-0,108	0,025
22	-0,320	0,258
23	-0,066	0,014

Table S2: Average coreness differences and their associated p-value. Ordered in descending $\bar{\mathcal{C}}_{\mathrm{HC},i}$. Significant values ($\alpha_{\mathrm{FDR}} = 0,025$) are in bold.

Label	$\bar{\mathcal{C}}_{\mathrm{AD},i}$	$\bar{\mathcal{C}}_{\mathrm{HC},i}$	$\bar{\mathcal{C}}_{\mathrm{AD},i} - \bar{\mathcal{C}}_{\mathrm{HC},i}$	p-Value
superiorfrontal R	0,923	0,952	-0,029	0,743
precentral R	0,883	0,921	-0,038	0,719
superiorfrontal L	0,846	0,921	-0,075	0,579
precentral L	0,695	0,916	-0,221	0,543
**superiortemporal R**	0,609	0,862	-0,253	**0,005**
middletemporal R	0,677	0,855	-0,178	0,121
superiorparietal R	0,781	0,806	-0,025	0,650
**lateraloccipital L**	0,562	0,794	-0,233	**0,020**
postcentral R	0,696	0,778	-0,082	0,613
**lateraloccipital R**	0,520	0,776	-0,256	**0,008**
inferiorparietal R	0,535	0,763	-0,228	0,071
fusiform L	0,575	0,720	-0,145	0,133
lingual L	0,558	0,715	-0,157	0,210
superiortemporal L	0,577	0,703	-0,126	0,316
**middletemporal L**	0,411	0,681	-0,270	**0,017**
lingual R	0,445	0,660	-0,214	0,034
fusiform R	0,494	0,650	-0,156	0,203
**inferiortemporal R**	0,344	0,641	-0,297	**0,002**
superiorparietal L	0,632	0,637	-0,005	0,992
precuneus R	0,598	0,631	-0,033	0,756
inferiortemporal L	0,478	0,569	-0,091	0,288
supramarginal R	0,482	0,513	-0,032	0,674
inferiorparietal L	0,401	0,502	-0,101	0,279
postcentral L	0,493	0,501	-0,009	0,787
precuneus L	0,296	0,437	-0,142	0,173
insula R	0,404	0,398	0,006	0,666
**pericalcarine L**	0,126	0,379	-0,254	**0,012**
rostralmiddlefrontal R	0,342	0,334	0,008	0,880
pericalcarine R	0,176	0,304	-0,128	0,151
insula L	0,369	0,290	0,079	0,388
supramarginal L	0,255	0,270	-0,015	0,771
lateralorbitofrontal L	0,223	0,254	-0,031	0,747
rostralmiddlefrontal L	0,299	0,213	0,086	0,349
cuneus R	0,091	0,191	-0,099	0,391
isthmuscingulate L	0,134	0,186	-0,052	0,324
posteriorcingulate L	0,116	0,172	-0,056	0,559
medialorbitofrontal L	0,226	0,158	0,067	0,221
medialorbitofrontal R	0,178	0,136	0,042	0,709
lateralorbitofrontal R	0,109	0,131	-0,022	0,696
isthmuscingulate R	0,086	0,122	-0,037	0,907
cuneus L	0,100	0,118	-0,018	0,201
posteriorcingulate R	0,174	0,115	0,059	0,192
caudalmiddlefrontal R	0,040	0,086	-0,045	0,966
**paracentral R**	0,151	0,076	0,074	**0,022**
parsopercularis R	0,010	0,057	-0,048	0,337
parsopercularis L	0,049	0,046	0,002	1,000
caudalmiddlefrontal L	0,047	0,041	0,006	0,946
bankssts R	0,018	0,033	-0,015	0,326
rostralanteriorcingulate L	0,032	0,031	0,001	0,568
bankssts L	0,010	0,021	-0,012	0,588
parahippocampal L	0,025	0,021	0,005	0,928
parstriangularis R	0,002	0,015	-0,014	0,754
paracentral L	0,036	0,015	0,021	0,462
parahippocampal R	0,035	0,014	0,021	0,679
parstriangularis L	0,007	0,010	-0,003	0,909
caudalanteriorcingulate L	0,014	0,009	0,004	0,629
caudalanteriorcingulate R	0,067	0,008	0,059	0,929
temporalpole L	0,008	0,007	0,001	0,805
rostralanteriorcingulate R	0,021	0,005	0,016	0,264
entorhinal L	0,004	0,005	-0,001	0,399
transversetemporal L	0,000	0,004	-0,004	0,189
frontalpole L	0,000	0,003	-0,003	0,368
parsorbitalis L	0,004	0,003	0,001	0,874
temporalpole R	0,010	0,002	0,008	0,682
parsorbitalis R	0,001	0,002	-0,002	0,630
transversetemporal R	0,001	0,002	-0,002	0,630
entorhinal R	0,000	0,000	0,000	NA
frontalpole R	0,000	0,000	0,000	NA
